# Income rank and depressive symptoms among employees in Germany − A 5-year cross-lagged panel analysis

**DOI:** 10.1016/j.ijchp.2024.100485

**Published:** 2024-07-10

**Authors:** Frank Euteneuer, Stefan Salzmann, Philipp Süssenbach

**Affiliations:** a Department of Psychology, Clinical Psychology and Psychotherapy, Medical School Berlin, Berlin, Germany; b Division of Translational Clinical Stress Research, Institute of Neuroscience and Biopsychology for Clinical Application, Medical School Berlin, Berlin, Germany; c Medical Psychology, Department of Medicine, Health and Medical University, Erfurt, Germany; d Division of Clinical Psychology and Psychotherapy, Philipps University of Marburg, Marburg, Germany; e Fachhochschule des Mittelstands (FHM) Bielefeld–University of Applied Sciences, Bielefeld, Germany

**Keywords:** Income rank, Income, Depressive symptoms, Cross-lagged panel, Longitudinal study

## Abstract

**Background/Objective:**

Socioeconomic disparities in mental health are well-established. Previous research suggests that relative income rank is associated with depressive symptoms above and beyond absolute income. This study aimed to investigate the predictive value of income rank for future depressive symptoms while accounting for absolute income. Exploring potential reverse pathways from depressive symptoms to income rank was a secondary objective.

**Method:**

A two-wave cross-lagged panel design with a 5-year follow-up was used to analyze data for income rank, absolute income, and two dimensions of depressive symptoms (i.e., cognitive-affective and somatic symptoms) from initially 4,201 employees. Income rank was calculated for reference groups, based on the same gender, the same 5-year age band, and the same occupational skill level.

**Results:**

Lower income rank at baseline predicted a higher severity of cognitive-affective depressive symptoms at five-year follow-up, even after adjusting for absolute income. In contrast, income rank did not demonstrate a significant unique longitudinal association with somatic depressive symptoms when simultaneously taking absolute income into account. There was no evidence for the assumption that depressive symptoms are predictive for future income rank (i.e., reverse pathway).

**Conclusions:**

Cognitive-affective symptoms of depression might be particularly responsive to social comparisons and a relatively low social rank.

## Introduction

Socioeconomic disparities in health are one of the most established patterns of social inequality (Adler & Ostrove, 1999; Pickett & Wilkinson, 2015). With regard to mental health, a socioeconomic gradient is well-established for depression, suggesting that individuals with lower socioeconomic status show higher levels of depressive symptoms and an elevated risk for depressive disorders (Dougall et al., 2023; Lorant et al., 2003; Pickett & Wilkinson, 2010). However, multiple pathways may elucidate the connection between socioeconomic status and depression. Similar to other health conditions, the inverse association between socioeconomic status and depression may partly arise from limited access to health-related factors (e.g., health care, protective environmental factors) and financial strain resulting from lower financial resources (e.g., low household income) (Dougall et al., 2023; Guerra & Eboreime, 2021; Matthews et al., 2010). In recent decades, however, several lines of research have argued that individuals relative socioeconomic ranks rather than absolute financial resources are important for health, particularly for the development of depressive symptoms in richer societies (Euteneuer, 2014; Gero et al., 2017; Hoebel et al., 2017; Hounkpatin et al., 2015; Pickett & Wilkinson, 2010; Schubert et al., 2016; Wetherall et al., 2015, 2019). Via the process of social comparison with others (Festinger, 1954), individuals develop a sense of their relative position in socioeconomic hierarchies (Adler et al., 2000; Cohen, 1999; Goodman et al., 2001; Hounkpatin et al., 2015). The perception of a lower rank in contrast to others, in turn, may influence physical and mental health through various psychological (e.g., negative emotions, need depletion, depressogenic cognitions) and stress-related biological mechanisms (Dougall et al., 2023; Euteneuer, 2014; Hoebel & Lampert, 2018; Matthews et al., 2010; Pickett & Wilkinson, 2015; Sapolsky, 2004; Schubert et al., 2016). From an evolutionary perspective, the argument has been put forward that depressive symptoms in response to a low social rank serve an adaptive function grounded on defensive shutdown strategies in animals. In this regard, low mood may be helpful for engaging in depression-like behaviors (e.g., social withdrawal and apathetic behavior) signaling a "no threat" status to dominants which may promote survival in competitive social situation (Gilbert, 2001; Gilbert & Allan, 1998; Price et al., 2007; Sloman et al., 2003; Wetherall et al., 2019; Wood et al., 2012).

Studies that have focused on rank-based socioeconomic aspects of health have utilized different approaches. A large amount of research in this field has assessed subjective social status (SSS) using a visual social ladder where individuals are asked to rank their socioeconomic or social position in contrast to others of a specific reference group (e.g., society of the country, community, school, workplace) (Adler et al., 2000; Cohen, 1999; Goodman et al., 2001). In several studies, SSS has been found to be related to poor health outcomes, including depressive symptoms and depressive disorders, above and beyond absolute objective measures of socioeconomic status such as income, education, and occupation (Cundiff et al., 2020; Euteneuer, 2014; Euteneuer et al., 2021; Hoebel et al., 2017; Hoebel & Lampert, 2018; Lange et al., 2023; Madigan & Daly, 2023; Pössel et al., 2022; Scott et al., 2014; Wetherall et al., 2019). At the same time, in another strain of research the relative socioeconomic rank, based on objective socioeconomic measures such as income and wealth, has been studied. These studies have calculated a measure of income rank, according to which the number of people within a reference group who have an income lower than that of the individual is compared to the total number of people within the individual's reference group (Boyce et al., 2010; Brown et al., 2008; Daly et al., 2015; Garratt et al., 2017; Hounkpatin et al., 2015, 2016; Macchia, 2023; Stewart et al., 2006). In terms of depression, this objective measure of income rank has also been found to be negatively related with depressive symptoms above and beyond absolute income in studies using cross-sectional (Gero et al., 2017) and longitudinal data (Hounkpatin et al., 2015).

In consideration of the above, the present study aimed to examine bidirectional longitudinal associations of an objective measure of income rank with depressive symptoms in a representative sample of employees in Germany using cross-lagged panel modeling with a five-year time lag. The present study aimed to extend the knowledge on associations between relative income rank and depressive symptoms in several regards. First, the present work extends longitudinal research on income rank and depression in U.S. and British individuals (Hounkpatin et al., 2015) by studying a German sample. Second, we specified a valid latent measurement model for depressive symptoms which allows us to study differential associations of income rank with two dimensions of depressive symptoms (i.e., cognitive-affective depressive symptoms, somatic depressive symptoms) (Patel et al., 2019). We hypothesized that a lower income rank is predictive for intensities of future cognitive-affective symptoms (Hypothesis 1) and somatic symptoms (Hypothesis 2) of depression, above and beyond the putative predictive value of absolute income. Third, an additional exploratory research question involved examining whether symptoms of depression have predictive value for future income rank (i.e., the reverse pathway). Although longitudinal associations between income rank and depressive symptoms have been examined in previous research (Hounkpatin et al., 2015), the existence of such reverse links is understudied. This point may be of relevance since poor mental and physical health can serve as a risk factor for downward social mobility and may thus also have predictive value for lower income and a decline in one`s social rank (Euteneuer et al., 2021; Nobles et al., 2013; Smith, 2004). Importantly, exploring a reverse pathway from depressive symptoms to income rank does not contradict social rank explanations for depression since the mechanisms involved in both pathways are not mutually exclusive.

## Method

### Participants and procedure

Data were obtained from the first wave in 2011/2012 (i.e., baseline) and the second wave in 2017 (i.e., follow-up) of the German Study of Mental Health at Work (S-MGA) (Scientific use file version 1, DOI: 10.48697/smga.w1w2.suf.1). The study design and sampling procedures have been extensively described elsewhere (Pattloch et al., 2021; Rose et al., 2017). The S-MGA is a representative survey study of the German working population, using randomly selected employees from social insurance data. From 13,590 sampled addresses at baseline, 4511 individuals agreed to participate, resulting in a response rate of 33.2 %. Of these initial sample, a total of 4201 individuals were employed when participating in the study. The S-MGA study has been approved by the ethics committee of the Federal Institute for Occupational Safety and Health. Informed consent was obtained from each participant. Data assessment was conducted according to the German data protection requirements.

### Absolute income and income rank

In the S-MGA questionnaire, nine categories for household net income per month were defined: (1) 1—<750€, (2) 750—<1000€, (3) 1000—<1500€, (4) 1500—<2000€, (5) 2000—<2500€, (6) 2500—<3000€, (7) 3000—<4000€, (8) 4000—<5000€, (9) ≥ 5000€. In each category the midpoint was set as the household net income of the study participants. A midpoint for category (9) was calculated by using a median-based Pareto curve estimate (Jargowsky & Wheeler, 2018; Parker & Fenwick, 1983). To equivalize household net income, it was divided by the square root of the household size (Dudel et al., 2020). The equivalized household net income variable was then log-transformed to receive a useful and established benchmark for absolute income against which to test income rank (Boyce et al., 2010).

People tend to compare themselves to others who are similar in various aspects (Goethals & Darley, 1977; Major & Forcey, 1985; Shah, 1998; Singer, 1981). To calculate income ranks for the present sample of employees, we thus used combined reference groups based on the same gender, the same 5-year age band (i.e., birth year intervals: 1951—1955, 1956—1960, 1961—1965, 1966—1970, 1971—1975, 1976—1980), as well as the same occupational skill level (i.e., unskilled workers, skilled workers, semi-professionals, academics/managers) according to the International Standard Classification of Education, which was based on the International Standard Classification of Occupations 2008 (Burr et al., 2021; International Labor Office, 2012). This procedure resulted in a total of 48 reference groups. Calculation of income rank was based on a well-established method (Boyce et al., 2010; Brown et al., 2008; Daly et al., 2015; Garratt et al., 2017; Hounkpatin et al., 2015, 2016; Macchia, 2023; Stewart et al., 2006). In this regard, a simple rank-based model was applied, according to which individuals compare themselves with other people in their reference group and assess whether each person within the same reference group earns more or less than they do. The number of people within the reference group who have an income lower than that of the individual (i – 1) is divided by the total number of people within the individual's reference group (n – 1). The resulting ratio provides an individual income rank normalized between 0 and 1.

### Depressive symptoms

Both waves included the nine-item German version (Löwe et al., 2002) of the Patient Health Questionnaire 9 (PHQ-9) (Kroenke et al., 2001) to assess the severity of depressive symptoms. Participants indicated, on a 0–3 scale ranging from “not at all” to “nearly every day”, the intensity of the following symptoms over the last two weeks: Items 1 (anhedonia), 2 (depressed mood), 3 (sleep disturbance), 4 (fatigue), 5 (appetite changes), 6 (low self-esteem), 7 (concentration problems), 8 (psychomotor disturbances), and 9 (suicidal ideation). Total scores range from 0 to 27, with higher scores indicating higher severity of depressive symptoms.

Valid latent modelling of longitudinal data requires measurement invariance (MI) across time to ascertain that the same construct is measured at each wave. Confirmatory factor analyses (CFAs) were thus performed a) to ensure factorial validity of the latent factor(s) for depressive symptoms and b) to confirm scalar measurement invariance across both waves. First, CFAs were conducted for baseline data from wave one. Because previous findings and theoretical assumptions support several valid models for the PHQ-9 (Boothroyd et al., 2019; Patel et al., 2019; Petersen et al., 2015), the most plausible measurement models were compared. Based on previous findings and theoretical assumptions, CFAs were conducted using a model with one latent factor (Model 1) (Boothroyd et al., 2019; Galenkamp et al., 2018; González-Blanch et al., 2018; Huang et al., 2006; Keum et al., 2018; Merz et al., 2011), and three models (Models 2A-2C) using two latent correlated factors. Model 2A specifies that items 1, 2, 6, 7, 8, and 9 load on a cognitive-affective factor, and that items 3, 4, and 5 load on a somatic factor (Chilcot et al., 2013; Doi et al., 2018; Krause et al., 2008; Patel et al., 2019). Model 2B is similar to model 2A, with the exception that item 8 loads on the somatic factor but not on the cognitive-affective factor (Baas et al., 2011; de Jonge et al., 2007; Reich et al., 2018). In model 2C, items 1, 2, 6 and 9 load on the cognitive-affective factor, while items 3, 4, 5, 7 and 8 are loading on a somatic factor (Boothroyd et al., 2019; Elhai et al., 2012; Petersen et al., 2015; Richardson & Richards, 2008). A large-sample study including 31,366 U.S. adults previously compared the above described models and found that all of these models demonstrate close model-data fit (Patel et al., 2019).

To be considered for further analysis, potential valid models had to show a) appropriate fit with respect to fit indices such as the comparative fit index (CFI), the root mean square error of approximation (RMSEA) and the standardized root mean square residual (SRMR), and b) minimum factor loadings (FL range) of 0.4 (Hair et al., 2010). In line with previous findings (Patel et al., 2019), all baseline measurement models were acceptable (Model 1: CFI= 0.916, RMSEA= 0.067, 90 % CI of RMSEA= 0.062–073, SRMR= 0.041, FL range= 0.484–.719; Model 2A: CFI= 0.933, RMSEA= 0.061, 90 % CI of RMSEA= 0.056–.067, SRMR= 0.038, FL range= 0.488–.749; Model 2B: CFI= 0.925, RMSEA= 0.065, 90 % CI of RMSEA= 0.060–.070, SRMR= 0.081, FL range= 0.481–.738; Model 2C: CFI= 0.932, RMSEA= 0.062, 90 % CI of RMSEA= 0.057–.067, SRMR= 0.038, FL range= 0.491–.755). Models 2A and 2C were superior to model 1, with a difference of ΔCFI ≥ 0.01 indicating substantially better fit (Cheung & Rensvold, 2002). Model 2A did show a numerical but not a meaningful better fit than model 2C (ΔCFI < 0.01). We therefore follow a theory-driven suggestion of Patel et al. (2019) and prefer model 2A over model 2C for further analyses. When applying model 2A for data from follow-up (i.e., wave 2), the model fit was also appropriate (CFI= 0.956, RMSEA= 0.053, 90 % CI of RMSEA= 0.046–060, SRMR= 0.032, FL range= 0.541–.784).

Next, CFAs were performed to test for scalar MI across both waves. For this purpose, three models with increasingly stringent restrictions were gradually compared (Meredith, 1993; Putnick & Bornstein, 2016). First, configural MI was evaluated which means that the same items were specified to measure the same constructs across both waves when both models (i.e., wave one and wave two) are tested simultaneously. The configural model was compared to a more constrained model to test for metric MI, which also requires equivalent factor loadings across waves. Finally, we compared the metric model with a model for scalar MI, which additionally requires the equivalence of item intercepts across waves. Because of the (over-) sensitivity of the χ^2^ -measure in large samples, MI models were evaluated based on the CFI, the RMSEA, as well as the SRMR. In accordance with established recommendations, changes across models in the CFI of less than −0.010, in the RMSEA of less than 0.015, and in SRMR of less than 0.010 were considered to ensure MI (Chen, 2007; Putnick & Bornstein, 2016). With respect to longitudinal MI, differences in fit indices between the configural model (CFI= 0.947, RMSEA= 0.035, 90 % CI of RMSEA= 0.033–.038, SRMR= 0.033), the metric model (CFI= 0.948, RMSEA= 0.034, 90 % CI of RMSEA= 0.032–.037, SRMR= 0.034) and the scalar model (CFI= 0.946, RMSEA= 0.034, 90 % CI of RMSEA= 0.031–.035, SRMR= = 0.034) revealed support for scalar MI.

### Statistical analysis

To test cross-lagged relations, we specified three cross-lagged panel models (CLPM) which include manifest variables for single-item variables and latent factors for cognitive-affective and somatic depressive symptoms. First, two separate models were run for income rank (Model A) and income (Model B) to examine bidirectional associations between each variable (i.e. income and income rank) and dimensions of depressive symptoms.

**Fig. 1:**
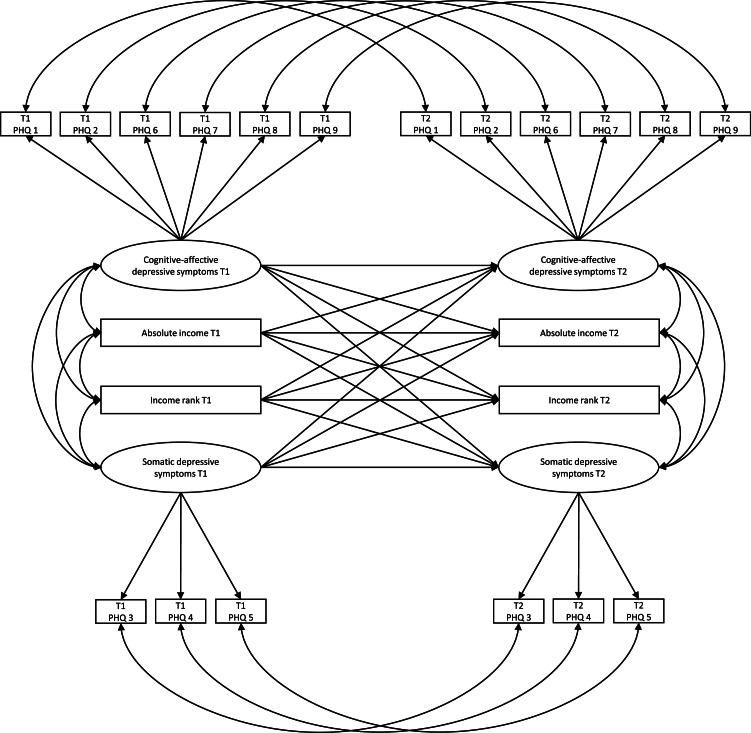
Specification of the main cross-lagged panel model (i.e., model c). For ease of presentation, covariates are not displayed. PHQ = Patient Health Questionnaire-9.

To address hypotheses 1 and 2, the main model (Model C) examined whether income rank has unique longitudinal associations with cognitive-affective and somatic depressive symptoms when simultaneously considering income. Fig. 1 illustrates the structure for this full model. Maximum likelihood estimations with robust standard errors were performed to use all available data from all participants (*n* = 4201) under the assumption that missing values were at random and to ensure robustness against multivariate non-normality. All models were adjusted for the most common theoretical covariates used in previous research in the field (e.g., Boyce et al., 2010; Daly et al., 2015; Hounkpatin et al., 2016; Macchia, 2023; Macchia et al., 2020; Hounkpatin et al., 2015), such as age (i.e., birth year intervals), gender, the occupational skill level as a combined measure for education/occupation (time-varying), marital status (time-varying), as well as the average income (i.e., reference income) of an individual's reference group (time-varying). Analyses were carried out with Mplus7 (Muthén & Muthén, 1998–2012). As directional effects were expected for all cross-lagged associations, one-tailed p-values are reported.

## Results

Table 1 shows descriptive statistics for baseline (2011/12) and five-year follow-up (2017). Data for the 4201 employees at baseline were available as follows: Birth year intervals (*n* = 4201; 100 %), gender (*n* = 4201; 100 %), occupational skill level education (*n* = 4188; 99.7 %), marital status (*n* = 4190; 99.7 %), absolute income (*n* = 3704; 88.2 %), income rank (*n* = 3693; 87.9 %), items for depressive symptom severity (*n* = 3795 – 3809; 90.3–90.7 %). In 2017, the follow-up wave, a total of 2481 completed the second interview (i.e., follow-up rate 59.1 %). Data for this wave were available as follows: occupational skill level education (*n* = 2198; 88.6 %), marital status (*n* = 2481; 100 %), absolute income (*n* = 2380; 95.9 %), income rank (*n* = 2108; 85.0 %), items for depressive symptom severity (*n* = 2344–2355; 94.5–94.9 %). Longitudinal models were based on all available data from all participants (*n* = 4201).

**Table 1:** Descriptive Statistics at baseline (*n* = 4201) and follow-up (*n* = 2481).

Variables	T1 (2011/12)	T2 (2017)
Birth year intervals, *n* (%)		
1951—1955	632 (15.0)	369 (14.9)
1956—1960	798 (19.0)	493 (19.9)
1961—1965	914 (21.8)	557 (22.5)
1966—1970	841 (20.0)	503 (20.3)
1971—1975	593 (14.1)	348 (14.0)
1976—1980	423 (10.1)	211 (8.5)
Gender, *n* (%)		
Female	2105 (50.1)	1271 (51.2)
Male	2096 (49.9)	1210 (48.8)
Occupational skill level, *n* (%)		
Unskilled workers	282 (6.7)	144 (5.8)
Skilled workers	1882 (44.7)	1037 (41.8)
Semi-professionals	1097 (26.1)	684 (27.6)
Academics/managers	927 (22.1)	611 (24.6)
Marital status, *n* (%)		
Married (including cohabitating)	3295 (78.6)	1988 (80.1)
Not married (single, divorced, widowed, separated)	895 (21.4)	493 (19.9)
Absolute income (€)^a^, mean (SD)	1897.24 (814.87)	2247.30 (908.32)
Income rank^b^	0.47 (0.29)	0.46 (0.29)
Patient Health Questionnaire-9, mean (SD)		
Total depressive symptom severity	4.33 (3.56)	4.55 (3.72)
Cognitive-affective depressive symptom severity	1.88 (1.93)	1.94 (2.06)
Somatic depressive symptom severity	2.45 (1.99)	2.63 (2.03)

*Note.* Values shown as mean (SD) for manifest variables unless otherwise noted.

a Household-size-adjusted net income. Untransformed values are presented for descriptive reasons. Models are based on log-transformed values.

b Income rank within a reference group of individuals with the same gender, the same 5-year age band, as well as the same occupational skill level.

Table 2 presents estimates and corresponding test statistics for all CLPMs. Model A which included income rank in the absence of absolute income indicated that a lower income rank at baseline predicts the severity of cognitive-affective depressive symptoms (β=−0.09, *p*<.001) and somatic depressive symptoms (β=−0.05, *p*=.028) at follow-up. With respect to a reverse depressive symptoms-to-income rank-pathway, no significant associations were observed. Model B which focused on absolute income in the absence of income rank indicated that also lower income at baseline predicts both the severity of cognitive-affective symptoms (β=−0.10, *p*<.001) and somatic symptoms (β=−0.07, *p*=.010) of depression at follow-up. The fit of the income rank model (AIC=131,487.75) was superior to the absolute income model (AIC=134,926.91). Therefore, income rank and income were significant predictors for both dimensions of depressive symptoms when entered as the only income-related predictor. However, in line with hypothesis 1, the main model (Model C) indicated that lower income rank is linked to cognitive-affective depressive symptoms above and beyond absolute income (AIC=125,322.44) when both income variables were entered simultaneously. Accordingly, individuals with lower income rank at baseline showed a higher intensity of cognitive-affective depressive symptoms (β=−0.13, *p*=.018) at follow-up, and absolute income accounted for no additional variance. Contrary to hypothesis 2, model C suggested that income rank has no unique negative cross-lagged longitudinal associations with somatic depressive symptoms when simultaneously considering absolute income. However, absolute income remained a significant predictor for somatic depressive symptoms (β=−0.19, *p*=.033) in the presence of income rank.

**Table 2:** Results of cross-lagged panel models: Standardized estimates with standard errors for bidirectional associations between income rank, income, as well as cognitive-affective and somatic depressive symptoms.

Model and predictor	T2 Income rank^a^		T2 Absolute Income^b^		T2 Cognitive-affective symptoms		T2 Somatic symptoms	
	β	SE	β	SE	β	SE	β	SE
Model A								
T1 Income rank^a^	0.56^c^	0.02			−0.09^c^	0.02	−0.05^e^	0.02
T1 Cognitive-affective symptoms	−0.10	0.07			0.47^c^	0.11	−0.06	0.11
T1 Somatic symptoms	0.06	0.07			0.10	0.11	0.64^c^	0.11
Model B								
T1 Absolute income^b^			0.59^c^	0.02	−0.10^c^	0.02	−0.07^e^	0.03
T1 Cognitive-affective symptoms			−0.14^d^	0.06	0.47^c^	0.11	−0.06	0.11
T1 Somatic symptoms			0.10	0.06	0.11	0.11	0.64^c^	0.11
Model C								
T1 Income rank^a^	0.35^c^	0.08	0.16^e^	0.07	−0.13^e^	0.06	0.11	0.08
T1 Absolute income^b^	0.24^d^	0.09	0.42^c^	0.09	0.05	0.07	−0.19^e^	0.10
T1 Cognitive-affective symptoms	−0.12^e^	0.07	−0.15^d^	0.06	0.47^c^	0.11	−0.07	0.11
T1 Somatic symptoms	0.05	0.07	0.10^e^	0.06	0.10	0.11	0.64^c^	0.11

*Note.* T1 = baseline (2011/12), T2 = follow-up (2017). All analyses are adjusted for age (i.e., birth year intervals), gender, occupational skill level, and the average income (i.e., reference income) of an individual's reference group.

a Income rank within a reference group of individuals with the same gender, the same 5-year age band, and the same occupational skill level.

b Log-transformed household-size-adjusted net income.

c = *P* < .001.

d = *p* < .01.

e *P* < .05.

## Discussion

The present work extends previous research by examining bidirectional longitudinal associations between income rank and depressive symptoms in a representative sample of German employees. As hypothesized, lower income rank at baseline predicted a higher severity of cognitive-affective depressive symptoms at five-year follow-up, even after adjusting for absolute income. Contrary to our hypothesis, income rank did not demonstrate a significant unique longitudinal association with somatic depressive symptoms. Of interest, we did not find evidence for the assumption that depressive symptoms are also predictive for future income rank (i.e., the reverse pathway).

The observation that lower income rank predicts the severity of cognitive-affective depressive symptoms is in accordance with longitudinal findings from US and British samples, showing that income rank is inversely related with future general mental distress (Wood et al., 2012) and depressive symptoms (Hounkpatin et al., 2015), as assessed by the Center for Epidemiologic Studies Depression Scale. The results of the present study also complement these findings by suggesting that the link between income rank and mental health is unidirectional as no cross-lagged effect of depressive symptoms on income rank was observed. In the present sample, however, income rank did not predict somatic depressive symptoms when simultaneously taking absolute income into account. Rather, the present findings are indicative of a dissociation regarding the predictive value of both income-related variables. Whereas income rank dominated was uniquely predictive of cognitive-affective depressive symptoms, income, on the other hand, was uniquely predictive of somatic depressive symptoms. The reason for this putative dissociation is not clear, in particular when taking previous studies into account that have shown that income rank relates to a broad range of health variables above and beyond absolute income, including those that involve somatic aspects (Daly et al., 2015; Hounkpatin et al., 2016; Macchia, 2023). When considering the present results in isolation, the conspicuous association between income rank and cognitive-affective symptoms, as opposed to absolute income, could suggest that cognitive-affective symptoms such as negative mood and depressogenic cognitions are particularly responsive to social comparisons and a relatively low social rank (Price et al., 2007; Schubert et al., 2016). Conversely, the unique association between lower absolute income and somatic depressive symptoms, which can be biased by other physical health factors (Nan et al., 2012), may reflect an effect of the material conditions and resources available to individuals (e.g., better access to healthcare, healthier living conditions) on somatic symptoms (Dougall et al., 2023). However, this explanation is speculative and more longitudinal studies on social inequality that considers the multidimensional structure of depressive symptoms (Fried & Nesse, 2015; Patel et al., 2019) are warranted.

Notwithstanding the strengths of the present study such as the cross-lagged panel design for studying putative bidirectional associations between income rank and depressive symptoms, as well as the differential latent modeling of cognitive-affective and somatic symptoms of depression, limitations need to be reflected. First, because we studied a representative sample of German employees, the generalizability to more diverse groups and other nations or cultures is an open question. For example, income inequality, a factor that may moderate the association between income rank and health (Macchia et al., 2020), is comparatively low in Germany. Possibly, longitudinal associations between income rank and mental health might be stronger in countries with higher income inequality or weaker in those with lower income inequality. Secondly, there are currently no established guidelines for interpreting effect sizes in CLPMs Typically, these effects tend to be notably smaller compared to those observed in cross-sectional studies, as cross-lagged effects control for the stability of the constructs under scrutiny and thus the predictive value of income-related variables can only account for changes in depressive symptoms that occurred within the time frame studied. While this is a common problem associated with CLPMs, awareness of this issue is important. (Adachi & Willoughby, 2015). Third, given the lack of longitudinal research on income rank and health, it is unclear whether a shorter or longer follow-up period than 5 years would have changed the strength of associations. Finally, we did not explore putative psychological intermediate factors between income rank and depressive symptoms. Thus, future studies with more waves including putative mediating factors (e.g., depressogenic cognitions, entrapment and defeat, negative affect) (Gilbert & Allan, 1998; Griffiths et al., 2014; Price et al., 2007; Schubert et al., 2016) may be promising.

To conclude, this study provides evidence for a prospective association between lower income rank and cognitive-affective symptoms of depression above and beyond absolute income. In contrast, income rank did not demonstrate a significant unique longitudinal association with somatic depressive symptoms when simultaneously taking absolute income into account. Finally, there was no evidence for the assumption that depressive symptoms are also predictive for future income rank (i.e., the reverse pathway). Future studies on socioeconomic status and depression should take income rank into account to gain a clearer picture of the social determinants of mental health.

## Declaration of competing interest

The authors declare that they have no known competing financial interests or personal relationships that could have appeared to influence the work reported in this paper.
